# Social context shapes facial dynamics: human and machine decoding of conversation topics

**DOI:** 10.1038/s41598-025-30403-9

**Published:** 2026-01-22

**Authors:** Prasetia Putra, Johanna Köchling, Jana Straßheim, Christophe Bousquet, Britta Renner, Harald T. Schupp

**Affiliations:** 1 Centre for the Advanced Study of Collective Behaviour, University of Konstanz, Konstanz, Germany; 2 Department of Psychology, University of Konstanz, Konstanz, Germany

**Keywords:** Conversation, Facial action units, Machine learning, Facial expression, Computational biology and bioinformatics, Mathematics and computing, Psychology, Psychology

## Abstract

Conversation is fundamental to the human species, with facial expressions playing a crucial role in establishing shared understanding within specific conversational contexts. We hypothesized that variations in conversation topics, differing in levels of tension and personal disclosure, would elicit distinct facial behavior dynamics. We assessed facial activity during two types of natural conversations with varying tension levels in triads of unacquainted individuals: "get-to-know-each-other" and “moral dilemma” discussions. Human observers classified the conversation type with 82.11% accuracy based on facial dynamics alone. Strikingly, a machine learning model using three facial action units (AUs 4, 6, and 12) during speech-free moments achieved comparable accuracy (82.14%). Further analyses revealed that the model relied on the temporal dynamics of these AUs to distinguish conversational contexts. These findings show that machine-based facial coding, coupled with deep learning, can infer conversational context from facial expressions, offering a scalable tool for analyzing natural social interaction.

**Supplementary Information:**

The online version contains supplementary material available at 10.1038/s41598-025-30403-9.

## Introduction

 Humans are highly social, with estimates suggesting that up to 70% of waking time is spent in social interactions^1, 2^. Social interaction and communication are fundamental to human cognition and socio-emotional development, shaping their abilities and behaviors^2, 3, 4^. At the core of these interactions lies dialogue and conversation, which serve as the foundation of social engagement^5, 6, 7^. Focusing on dialogue has been transformative in language research, calling for a dyadic perspective to capture the inherently relational and interactive nature of language within conversations^5, 6, 7, 8^. In previous research, rules of conversation have been discovered, revealing a fast-paced system with exquisite timing of turn-taking, including a one-second window that differentiates between early, timely, and delayed responses, and the use of a filler word sounding like ‘huh’ to signal problems in conversations^9, 10^.

Crucially, verbal exchange is embedded in a continuous stream of visual and bodily cues, among which the face plays a central role^11, 12^. From infancy, humans are attuned to facial signals that guide attention and foster connection^13, 14^. By adulthood, facial behavior becomes a flexible, context-sensitive tool for conveying emotional meaning and managing conversational pragmatics^15, 16, 17, 18^. The Facial Action Coding System (FACS) offers a systematic method for quantifying expressions via Action Units (AUs). Smiling, one of the most studied expressions in interaction^19, 20, 21^, typically involves AU 12 (lip corner puller) and often AU 6 (cheek raiser) in Duchenne smiles, which are linked to genuine positive affect and social signals like friendliness and agreement^22, 23, 24, 25^. In contrast, AU 4 (brow lowerer) has been associated with negative affect such as anger or confusion and is often observed during disagreement, cognitive load, or moral evaluation^26, 27^. Electromyographic (EMG) studies support these links, showing reliable activity in the corrugator supercilii (AU 4) and zygomaticus major (AU 12) during negative and positive affective experiences, respectively^26, 28^. In conversation, these AUs are flexibly deployed not just to express emotion but to shape interaction—for instance, reinforcing shared understanding or signaling confusion^16, 29^.

The role of facial action units in conveying both emotional and pragmatic signals aligns with theoretical frameworks that conceptualize facial expressions as communicative and context-sensitive behaviors. The behavioral ecology view proposed by Fridlund^30^, Barrett’s theory of socially constructed emotion^31^, and Jack and Schyns’ work on the dynamic perception of facial signals^32^ all challenge the notion of a fixed one-to-one mapping between facial expressions and discrete emotions. Instead, these frameworks suggest that facial behavior functions as a flexible system shaped by contextual factors and interactional goals. We propose that social interactions unfold within a shared framework of understanding regarding the appropriate use of facial expressions across different conversational settings. Just as individuals adapt their language depending on whether they are in a professional or familial context, specific norms also shape facial expressions according to the social situation. Accordingly, we hypothesize that facial expressions systematically vary across conversational contexts, and that both human observers and machine learning algorithms can reliably detect these context-dependent patterns.

To test our hypothesis, we focused on two types of real-world conversations that differ markedly in social function and emotional tone: “get-to-know-each-other” conversations and “moral dilemma” discussions. In both conditions, participants were unacquainted with one another, allowing us to examine how facial behavior is shaped by situational context rather than prior relational dynamics. In the "get-to-know-each-other" condition^33^, participants engaged in informal, affiliative conversation, discussing topics such as hobbies, interests, and personal backgrounds. These interactions typically foster rapport, involve low-stakes self-disclosure, and reflect a socially open-ended format in which participants have considerable latitude in how they present themselves. Such interactions are common in everyday life — at workplaces, schools, or social gatherings — and play a key role in enabling humans to form new social bonds and coordinate with unfamiliar others^34, 35^. In contrast, the “moral dilemma” condition required participants to debate whether a terrorist should be tortured to prevent a deadly city-wide attack, creating a tension-filled, evaluative context reflective of political or organizational discourse. These two scenarios were selected to contrast conversational contexts along dimensions of affect, alignment, and communicative stakes.

To investigate whether facial behavior alone — stripped of linguistic content — conveys structured information about interactional context, we adopted a dual-method approach. First, we asked naive human observers to classify the type of conversation from muted video recordings. This behavioral task capitalizes on the human capacity to extract meaning from subtle nonverbal cues and provides a socially informed benchmark for what can be inferred from facial activity in real-world interactions^36^. Second, we applied a machine learning approach that leverages the temporal dynamics of facial behavior over the course of an interaction. Continuous time-series data for AU 4, 6, and 12 were converted into image-based representations and submitted to a pre-trained convolutional neural network (CNN) for binary classification of conversation type.

Further analyses were conducted to examine the contribution of temporal information to classification performance. First, we tested whether static differences in facial activity could account for classification outcomes by extracting time-agnostic features (e.g., mean and variance of AU signals) and training logistic regression (linear) and random forest (non-linear) models. Second, to assess the importance of temporal structure, we shuffled the AU time series — while preserving the overall signal distribution — before submitting them to the CNN. Finally, we varied the length and position of input segments (beginning, middle, or end of the conversation) to assess how temporal resolution and conversational phase affect model performance. Together, these analyses clarified the specific contribution of dynamic facial patterns to conversation-type classification.

## Methods

### Participants and data collection

The ethical committee of the University of Konstanz (No. 24/2020) approved the experimental procedure in accordance with the Declaration of Helsinki. All methods were carried out in compliance with approved guidelines. Participants provided informed consent and were debriefed after the experiment.

A total of 84 participants (69.05% female, M = 24.04 years, SD = 7.37), organized into 28 triads, were randomly assigned to one of two conversation topics: “get-to-know-each-other” or “moral dilemma.” Conversations were conducted via Zoom 5.6.5 [https://www.zoom.us] and lasted approximately 15 minutes. Each session was recorded and stored as a 25 fps mp4 video file. All participants completed pre- and post-experiment questionnaires. Some sessions included lunch delivery as part of a manipulation, not relevant to the present analysis. Effects of eating on social interaction behavior are reported in Straßheim and colleagues^37^.

In the "get-to-know-each-other" condition, participants engaged in casual conversation, discussing hobbies and personal interests. In the “moral dilemma” condition, participants debated whether it is acceptable to torture a terrorist to prevent an imminent attack. These two contexts differed in affective tone, stakes, and social alignment.

### Human classification of conversation topics

We conducted a behavioral study to assess whether naive observers could classify the recorded conversations based on facial behavior alone.

### Participants

Nineteen independent raters (18 females, M = 24.89, SD = 5.22) took part in the study. Raters were members of the same cultural background as the conversation participants. For reasons of data protection, student assistants and researchers at the lab, who had all signed a confidentiality agreement and were authorized to view the video material of the study, were recruited as raters. They were not involved in the original study or the design of this specific analysis and were authorized to view the muted video material.

### Stimuli and procedure

Raters viewed muted video excerpts of the 28 triadic conversations. Videos displayed both head and upper body posture. Each rater evaluated five randomly assigned videos, with the only restriction being that every video was rated at least three times. This resulted in a total of 95 human classification ratings.

Ratings were collected using Qualtrics (2023) [https://www.qualtrics.com]. Raters made a binary judgment (“get-to-know-each-other” vs. “moral dilemma”), indicated their confidence in their classification on a 6-point Likert scale, and used a visual analog scale to report when during the video they made their decision. Open-text fields allowed for free-form explanations.

### Data analysis

Accuracy was evaluated at the rating, video, and rater level. In addition to overall accuracy, we computed the Matthews Correlation Coefficient (MCC)^38^, which accounts for both true and false classifications, and Fleiss’ Kappa for inter-rater reliability^39^. Confidence and decision timing were compared for correct vs. incorrect ratings using independent-sample t-tests. All analyses were performed in R 4.3.3 [https://www.r-project.org].

### Machine Learning-Based classification of conversation topics

**Fig. 1:**
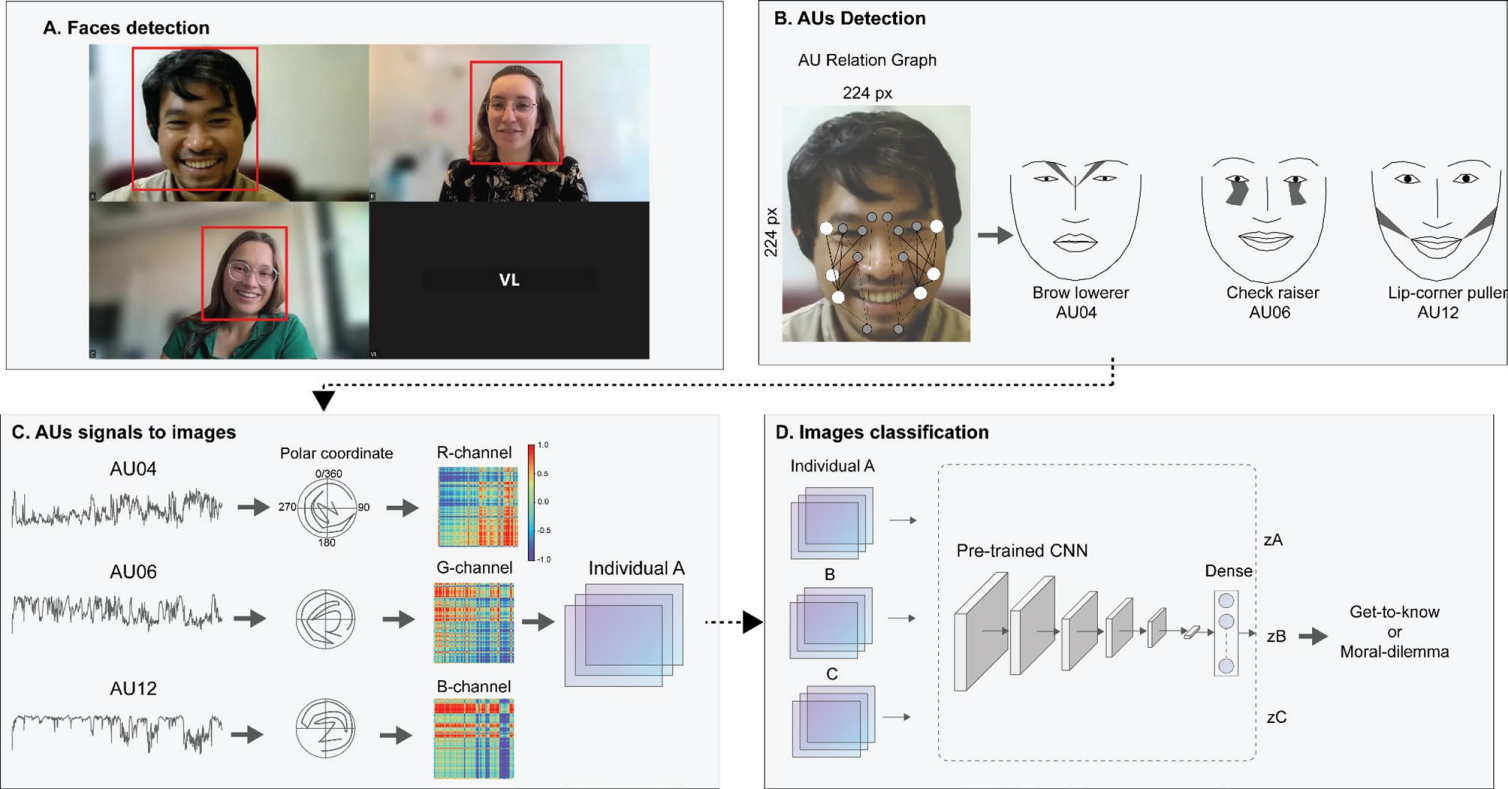
Framework for machine-learning-based coding of facial action units and deep learning binary classification of conversation topics. (**A**) In the first step, the face of each individual was detected. (**B**) In the second step, each frame was processed through ME-GraphAU to estimate the activity of specific facial action units (AUs): AU 4 (‘brow lowerer’; m. corrugator supercilii, m. depressor supercilii), AU 6 (‘cheek raiser’; m. orbicularis oculi), and AU 12 (‘lip-corner puller’; m. zygomaticus major). Their actions are illustrated using PyFeat software. (**C**) In the third step, the Gramian Angular Field technique was applied to convert AU time series data into images, where each pixel represents the correlation between two points in the time series. (**D**) This conversion enabled the analysis of time-series data using image-based deep learning techniques. A pre-trained convolutional neural network was then used for binary classification to determine the conversation topic. The faces shown in the graph are of the authors and lab members who did not participate in the experiment, all of whom provided written consent for their images to be displayed in this figure and published in an online open-access journal.

We then analyzed facial activity from the same recorded triadic conversations using a machine learning pipeline that detects faces, extracts facial action unit (AU) time series, converts them into image representations, and performs binary classification using deep learning techniques (Fig. 1). Analyses were performed in Python 3.9.19 [https://www.python.org*].*

### Face detection

In a first step, the original Zoom video recordings were pre-processed and cut into three separate videos containing only data from each of the three participants. Each of the videos was then submitted to RetinaFace, a state-of-the-art face detection algorithm, which achieved high performance (average precision equal to 91.4%) rates on the WIDER FACE hard test databases^40^. We then checked the quality of face detection. For 82 out of the 84 participants, the data analysis was conducted by setting the prediction score threshold at > = 0.99. For the remaining two videos, lower certainty scores were needed, which were derived by manual inspection of the data. Out of 1.890.210 images, 12.429 (0.66%) of the images had to be removed from analysis.

### Action unit coding

The coding of action unit activity was accomplished by employing AU Relation Graph (ME-GraphAU) which extracts the activity of AUs 1, 2, 4, 6, 7, 10, 12, 14, 15, 17, 23, and 24^41^. ME-GraphAU was selected by considering its benchmark performance, which scores the highest in BP4D and DISFA, and the availability of its pre-trained models. As a preprocessing step required by the pretrained model, the sequence of face images was resized into a 224 × 224 matrix before being submitted to the ME-GraphAU algorithm. Since the pre-trained Convolutional Neural Networks we used were limited to three channels (RGB), we selected AUs 4, 6, and 12—the most relevant for social interactions—as our inputs^22, 23, 24, 26^.

### Speech detection and signal masking

Since ME-GraphAU was trained exclusively on images of non-speaking participants, we limited our analysis to non-speaking periods to test the hypothesis that facial activity differs across conversation topics. High resolution of speech detection was obtained using Audacity 3.3.1 [https://www.audacityteam.org] (threshold level = −30.0 dB, threshold measurement = RMS level; minimum silence duration = 0.2 s, minimum label interval = 0.8 s, maximum leading silence = 0 ms, and maximum trailing silence = 0 ms) and the function Label Sounds providing millisecond resolution of speech activity by each individual. All identified sounds were manually verified as speech and differentiated from background noise (e.g., falling objects). Furthermore, laughter and filler sounds were scored separately and not included in the analysis.

In triadic conversations, where participants took turns speaking with pauses in between, approximately three-quarters of the total triadic interaction time was speech-free. The proportion of these silent periods did not significantly differ between the “get-to-know-each-other” (76.38%) and “moral dilemma” (77.71%) conditions, as indicated by a Kolmogorov–Smirnov test (D statistic = 0.21, *p* = 0.29). For the classification analysis, speech periods were masked in the AU signals by replacing the original values with zeros before converting the data into images, ensuring that only speech-free facial activity contributed to the conversation topic classification.

### Conversion to image representations

Taking advantage of pre-trained Convolutional Neural Networks (CNNs), we converted the AUs time-series into images using the Gramian Angular Field (GAF) method^42^. GAF represents the temporal correlation within different time intervals by calculating the trigonometric difference between each point of a signal. It preserves temporal dependency by representing the relation between each point with regard to the time interval. Transforming time-series data $\:X\:=\:\left\{{x}_{1,\:}{x}_{2,\:\cdot\:\cdot\:\cdot\:\cdot\:,\:}{x}_{N}\right\}$ using GAF involves three steps. First, X is standardized so that the values fall into the range of −1 to 1. Then, the normalized $\:\widehat{X}$ is transformed from Cartesian into Polar coordinates by encoding the value as angular cosine and the timestamp as the radius, which can be expressed as follows:

$$\:{\varPhi\:}_{i\:}=\:arccos\left(\widehat{{x}_{i}}\right),\:-1\:\le\:\widehat{{x}_{i}}\le\:1,{\widehat{x}}_{i}\in\:\widehat{X}\:$$

$$\:{r}_{i\:}\:=\:\frac{{t}_{i}}{N},\:{t}_{i}\in\:\:N$$

Lastly, the Gramian Angular Difference Field (GADF) is constructed by computing the trigonometric difference between each point using the following formula:

$$\:GADF\:=\:sin\:({\varPhi\:}_{i}\:-\:{\varPhi\:}_{j})$$

The values of GADF range from − 1 to 1, where negative values indicate a phase decrease (opposing signal behavior), and positive values indicate a phase increase (aligned signal behavior). The magnitude of the GADF reflects the strength of the phase change in the signal.

### Classification pipeline

Binary classification was conducted using a combination of pre-trained CNNs and a feed-forward neural network, implemented in PyTorch 2.1.1 [https://pytorch.org]. The aim was to determine whether the group’s discussion topic fell under the category of “get-to-know-each-other” (0) or “moral dilemma” (1). To match the input dimension of the pre-trained network, the RGB images of AU signals were resized to 224 × 224 before being fed into the model.

### Training and evaluation procedure of the machine learning model

The same optimal hyperparameter values obtained using the Grid Search algorithm were used for all experiments (see Code for details). The model was trained and validated using 5-fold cross-group validation, with a training set of 70%, validation set of 10%, and test set of 20%. This approach ensured that data from the same triad did not appear in both the training and test sets, enabling a robust evaluation of the model’s generalization ability to unseen conversation groups. During training, we computed binary categorical entropy loss to monitor the training and validation results, and to perform early stopping.

Confidence intervals (CIs) for model accuracy and MCC were computed using a value of 2.05 (*p* < 0.05). For the Area Under the Curve (AUC), CIs were estimated using a U-statistics approach^43^.

### Control analyses

To evaluate whether classification performance was driven by meaningful temporal dynamics rather than superficial patterns, we conducted three control analyses.

First, we tested whether the timing of speech alone could account for classification by replacing action unit values with binary indicators of speech presence (speech vs. non-speech).

Second, to assess the role of temporal structure, we randomly permuted the time series of each action unit while preserving the overall signal distribution.

Third, we tested whether static differences in facial activity alone could distinguish between the two conversation contexts. To do this, we extracted eight descriptive statistical features from the entire conversation recordings for action units (AUs) 4, 6, and 12: (1) maximum intensity, (2) mean activity, (3) standard deviation, (4) maximum duration of episodes (i.e., number of frames), (5) average duration of episodes, (6) minimum duration of episodes, (7) number of episodes, and (8) total number of frames during which the AUs were activated. These features were used to train logistic regression and random forest classifiers to perform binary classification (“get-to-know-each-other” vs. “moral dilemma”). We did not normalize the features, as normalization impaired the performance of the logistic regression classifier. The models were trained and validated using 5-fold cross-group validation on the same population as the proposed deep learning model. Further methodological details and implementation specifics of the control analyses can be found in the Supplementary Information.

## Results

### Human classification of conversation topics

**Fig. 2:**
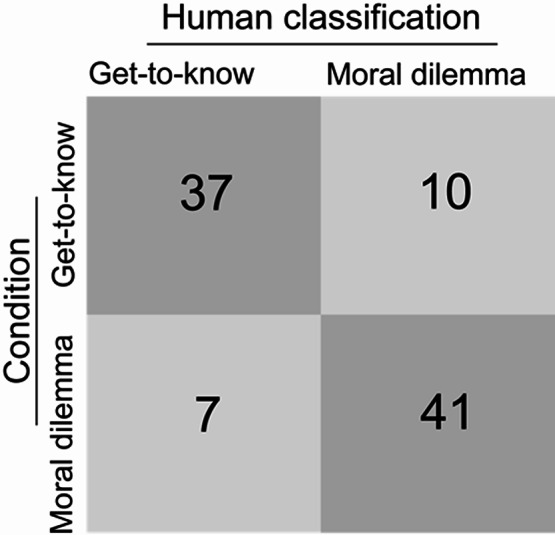
Classification performance of human raters. Confusion matrix displaying the proportion of correct and incorrect ratings (*N* = 95) for the two conversation topics.

Out of 95 total classifications, 78 were correct, yielding an overall accuracy of 82.11%. Accuracy was slightly higher for the “moral dilemma” (MD) conversations than for the “get-to-know-each-other” (GTK) topic (see Fig. 2), although this difference was not statistically significant (χ²(1) = 0.724, *p* = 0.395). In addition to accuracy, the Matthews Correlation Coefficient (MCC), which accounts for both true and false classifications, was 0.64, indicating very good classification performance^38^.

Next, differences in classification accuracy between the raters were examined. Eight out of 19 raters (42.11%) correctly classified all conversations, and seven raters (36.84%) made only one error. Two raters, however, correctly classified fewer than half of the conversations. Excluding these two raters would increase the overall accuracy of the human ratings from 82.11% to 87.06%.

The ratings were given with high confidence, averaging M = 4.55 on a 6-point rating scale, with no significant difference between conversation topics (M_GTK_ = 4.53, SD_GTK_ = 1.3; M_MD_ = 4.56, SD_MD_ = 1.01, t(93) = −0.128, *p* = 0.90). As expected, rater confidence was significantly higher for correctly classified conversations (M = 4.77, SD = 0.99) compared to those incorrectly classified (M = 3.53, SD = 1.33; t(93) = −4.378, *p* < 0.001, d = −1.17).

Participants indicated the point at which they made their classification decision on a time scale (1–100), with most decisions occurring within the first third of the conversation (M = 19.33, SD = 18.07). The timing of decisions did not significantly differ between the two conversation topics (M_GTK_ = 18.87, SD_GTK_ = 19.95; M_MD_ = 19.77, SD_MD_ = 16.21, t(93) = −0.241, *p* = 0.81). Consistent with confidence ratings, decisions for correct classifications were made earlier (M = 17.53, SD = 16.98) than for incorrect classifications (M = 27.59, SD = 21.02; (t(93) = 2.119, *p* = 0.037, d = 0.57). In addition, there was an inverse relationship between rater confidence and decision time (*r* = −0.682, *p* < 0.001).

Finally, classification accuracy varied between triads. Over half of the conversations (53.57%) were correctly classified by all 3–4 raters. However, the calculation of the Fleiss Kappa, performed separately for conversations with three and four raters (Kappa = 0.423 and 0.545, both *p* < 0.001) indicated moderate inter-rater agreement. Four conversations — three from the “get-to-know-each-other” condition and one from the “moral dilemma” condition - proved challenging, with only one rater correctly classifying them.

### Machine-based classification of conversation topics

**Fig. 3:**
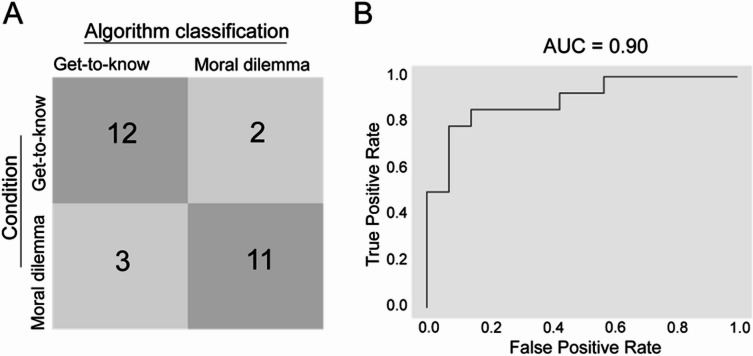
Classification performance of the machine learning model. (**A**) Confusion matrix displaying the proportion of correct and incorrect classification for the two conversation topics (*N* = 28). (**B**) Display of the ROC AUC curve.

We first evaluated the performance of our machine learning algorithm by calculating the accuracy. This resulted in an accuracy of 82.14% (95% Confidence Interval [CI] = [80.14%, 84.14%]). Inspection of the confusion matrix (Fig. 3A) revealed that two “get-to-know-each-other” conversations and three “moral dilemma” conversations were misclassified.

Second, as shown in Fig. 3B, we computed the area under the receiver operating characteristic curve (ROC AUC), which plots the true positive rate (i.e., sensitivity or recall) against the false positive rate (i.e., 1 - specificity). Ranging from 0 (worst result) to 1 (perfect result), our model achieved an AUC score of 0.90 (95% CI = [0.89, 0.91]), indicating excellent classification performance.

However, both accuracy and AUC scores can overestimate classification performance by not accounting for precision and negative predictive value. To address this, we calculated the Matthews correlation coefficient. Our model achieved an MCC score of 0.64 (95% CI = [0.59, 0.69]), confirming very good overall classification performance.

### Control analyses

Our machine learning model appears to classify conversation topics based on AU activity during the conversation, however, it is unclear whether this classification is driven by genuine patterns or spurious signals. The reported findings were based on participants’ AU data from speech-free segments, aligning with the training conditions of the ME-GraphAU algorithm. Notably, classification performance of the proposed model was poor when using the full conversation, with an accuracy of 57.14% (95% CI = [54.14%, 60.14%]) and an MCC score of 0.17 (95% CI = [0.11, 0.23]). However, differences in speech timing between the two conversation topics could influence the classifier’s performance. To rule out the possibility that the classifier relied on superficial patterns of speech versus speech-free segments, we replaced action unit activity with binary values, zeros for speech and ones for speech-free segments. The resulting accuracy of 50% (95% CI = [47.00%, 53.00%]) and MCC of 0.0 (95% CI = [−0.06, 0.06]) indicate that the timing of speech activity alone could not differentiate between conversation topics.

The GADF transformation enabled the DNN model to recognize temporal patterns, relationships, and sequences of facial activity in a spatially structured format, allowing it to leverage spatial feature extraction methods to detect trends and patterns over time. This approach is based on the premise that the dynamics of AU activity are crucial for accurate classification; thus, disrupting the temporal structure of AU activity is expected to impair classification performance. In a control analysis where each AU’s activity was randomly permuted while retaining speech-free segments, the classifier’s performance dropped significantly, achieving only 57.14% (95% CI = [54.14%, 60.14%]) accuracy and an MMC of 0.14 (95% CI = [0.07, 0.21]). These findings suggest that preserving the temporal dynamics of AU activity is essential for successful classification.

One could argue that an overly complex approach is being used for a relatively straightforward task. The two conversation topics may differ in the frequency of emotional or pragmatic facial expressions resulting in condition differences that can be captured through descriptive statistical measures. Accordingly, eight descriptive measures including mean and standard deviation (see Methods for more details) were used to train logistic regression (linear) and random forest (non-linear) algorithms to predict conversation topics in the same way as the proposed deep-learning-based approach. Classification performance was low for both models (accuracy = 60.71% with 95% CI = [57.71%, 63.71%] and 42.86% with 95% CI = [39.86%, 45.86%], MCC = 0.22 with 95% CI = [0.16, 0.28] and −0.15 with 95% CI = [−0.21, −0.09] for logistic regression and random forest models, respectively) providing no support for this hypothesis.

### Temporal and granularity effects on machine classification of conversation topics

**Fig. 4:**
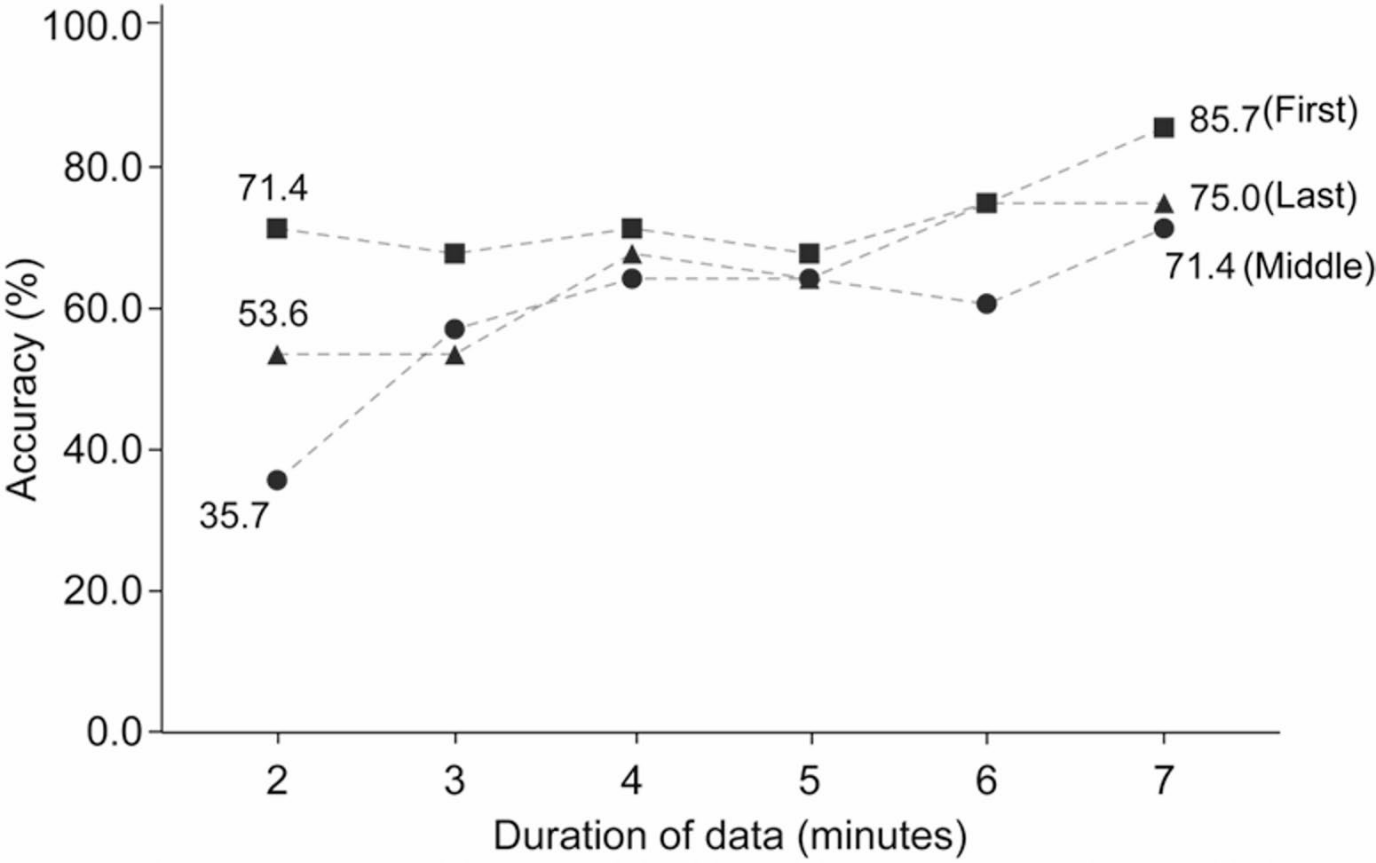
Accuracy rate of the model using different segments of data as input. The terms “First,” “Middle,” and “Last” indicate the specific time segments of the conversations from which the data were extracted. For instance, “2-First” refers to a 2-minute segment taken from the beginning of the conversation, “2-Middle” refers to a two-minute segment centered around the midpoint, and “2-Last” refers to the final 2-minute segment of the conversation.

Refined analyses enable a deeper understanding of how temporal factors and data granularity influence classification accuracy. The face may play a particularly significant role at the beginning of conversations, where establishing common ground — often referred to as “breaking the ice” — is crucial. Moreover, previous research has demonstrated that impressions and evaluations of others’ affective and personality traits can be formed from thin slices of behavior^44, 45^. To investigate these aspects, we varied the segment length of the data from 2 to 7 min and drew these segments from different time points in the conversation: specifically, the beginning, mid-point, or end. As shown in Fig. 4, three key findings emerged: First, classification performance varied based on the time point of the conversation, with segments from the beginning yielding higher classification accuracy than those from the middle or end. Second, even a two-minute segment from the beginning of the conversation exceeded the chance level of group classification (71.43%; 95% CI = [69.43%, 73.43%]). Third, and most importantly, using the first seven-minute segment resulted in excellent classification performance (85.71%; 95% CI = [83.71%, 87.71%]), slightly surpassing the accuracy achieved when using the entire conversation.

## Discussion

In conversations, interlocutors collaborate to create and maintain shared understanding, with social context playing a key role. For example, when comparing a business meeting to a casual chat among friends, formal, precise language is typically used in the former, while the latter features more casual and emotionally expressive speech. These contextual differences extend beyond words to include nonverbal signals. Conversations are shaped by social norms shared by participants, influencing both verbal and nonverbal behavior. While these distinctions may seem intuitive, they warrant empirical investigation, especially when social contexts differ subtly.

We used conversations between non-acquainted individuals as a model, allowing us to manipulate the topic’s relevance in terms of tension, seriousness, and personal disclosure while controlling for prior interpersonal experience. To evaluate how interpretable facial behavior is under these conditions, we asked naive observers to classify conversation types from muted video recordings. This task leveraged humans’ ability to extract meaning from subtle nonverbal cues and served as a socially grounded benchmark for what facial activity conveys in natural interactions. Observers from the same cultural background as the participants achieved high classification accuracy. This likely reflects (i) a shared understanding of facial and bodily expressions rooted in socio-cultural norms; (ii) topic-specific communicative and paralinguistic strategies; and (iii) the human ability to flexibly interpret facial expressions according to the demands of different social contexts.

Importantly, these results were obtained in the context of triadic conversations, a format that poses greater interactional complexity than dyads. Most everyday conversations involve two or three people^46, 47^, but the shift from dyadic to triadic interactions fundamentally alters the mechanics of communication. Coordinating turn-taking, determining who speaks when, and providing feedback through backchannels are all more demanding in triads. Moreover, the presence of an additional listener can modulate the willingness to disclose personal information due to increased social judgment^48^. Thus, any facial activity related to topic-specific social context must be interpreted in light of the baseline challenge of navigating triadic coordination. In this respect, the high classification accuracy underscores the remarkable human capacity to use facial expressions as a flexible and effective tool for managing complex social interactions.

Reliance on human judgments to study facial activity in natural conversations is challenging. Manual coding of facial action units is prohibitively time-consuming, requiring an estimated 115 days for the current dataset alone (https://www.erikarosenberg.com/more-about-facs), and human rating studies are similarly labor-intensive, especially when analyzing fine-grained conversational segments. This challenge can be addressed using automated analysis of facial action units (AUs) combined with machine learning. Machine learning offers scalable tools for processing large datasets. For instance, some studies have analyzed millions of images and videos to identify prototypical emotional expressions worldwide^49, 50^. Others have examined facial dynamics in conversation to capture interindividual differences in bonding, negotiation, and therapeutic communication^51, 52, 53, 54^. Uniquely, our study uses machine learning to test the hypothesis that facial behavior varies systematically with social context. This focus on the dynamics of facial activity was captured by our deep learning classifier containing not only the original time series of the action units but also their temporal correlations across time. The model accurately classified 82.14% of conversations during speech-free periods, matching human classification performance. These findings align with theoretical frameworks such as the behavioral ecology view of Fridlund and Barrett’s theory of socially constructed emotion that emphasize the flexible and context-driven nature of facial signals^30, 31^.

We then investigated whether the high classification performance depended on the temporal dynamics of facial activity or reflected differences in the engagement of action units associated with emotional signals (e.g., the frequency of smiles affecting AUs 6 and 12) and pragmatic signals (e.g., questioning and disagreement, increasing activity of AU 4) between topics. To address this, we disrupted the temporal sequence of action unit activity through data shuffling while preserving the overall statistical distribution. When dynamics were scrambled, classification dropped close to chance levels. This confirmed that classification success depends on the temporal dynamics of AUs 4, 6, and 12. Supporting this conclusion, classification based on summary statistics of AU activity (e.g., mean, standard deviation, activation duration) performed 20% worse than approaches leveraging full temporal information. These results align with evidence that conversational facial behavior derives its meaning not from individual AUs but from structured, context-sensitive combinations of facial signals, which can produce Gestalt-like shifts in interpretation^55^; and that map onto distinct social actions through their precise timing^56^. These findings may explain why our classifier depends on temporal dynamics: conversation type appears to be encoded in the coordinated, evolving interplay of multiple AUs rather than in their overall magnitude and frequency.

Effective dialogue involves both focal alignment, moment-to-moment coordination, and global alignment that develops over time^6^, see also^57^. The face, as part of a multimodal system, may offer preliminary insights into understanding this issue. Analyzing segments of conversations that differ in length and position within the interaction (beginning, middle, or end) can serve as a proxy for focal alignment. Interestingly, classification for ≤ 4-minute segments from the beginning of conversations exceeded chance level (~ 71%). Shorter conversation segments allow for a micro-analysis of the conversation, focusing on local alignment and context-dependent adjustments. Although short segments sufficed for topic classification, longer segments capturing global alignment yielded higher accuracy.

One might assume that more data leads to better generalization and performance. Yet, our findings show that 7-minute segments from the beginning outperformed full-length conversations. Notably, superior performance for segments taken from the beginning was a consistent finding across different segment lengths, possibly highlighting the unique nature of initiating a conversation — often metaphorically described as “breaking the ice” — during which a shared understanding is established. This aligns with human rating data, indicating that the conversation topic was identified within the first third of the interaction. Future research should test the hypothesis that facial cues are particularly informative at the start of conversations across a broader range of interaction contexts.

### Limitations

As a proof-of-principle, our findings highlight three key aspects of facial behavior in context: (i) classification fails when temporal dynamics are disrupted, showing that timing is critical; (ii) accuracy with short segments suggests that socially relevant facial behavior is temporally concentrated; and (iii) the need for all three AUs supports their combined role in conveying affective and pragmatic signals. Our control analyses show that performance relies on the temporal dynamics of AUs 4, 6, and 12, but the model does not reveal which patterns drove its decision. This limits insights into interpretability and feature importance. Future work should integrate explainable AI techniques (e.g., relevance propagation, saliency mapping, attention-based models) and fine-tune the pretrained CNN with additional data to enable robust interpretation of which facial signals carry context-relevant information. These approaches will support a shift from predictive power toward theoretical understanding.

Similar limitations arise when individuals are asked to report the cues underlying their social perceptions. Classic research in personality and interpersonal perception (e.g., Ambady & Rosenthal^45)^ shows that, while such judgments can be accurate, they typically rely on tacit, unconscious processing of multiple, distributed cues. It may be informative to assess whether trained experts in facial coding or social interaction (e.g., clinicians, therapists, or mediators) perform better at classification than naive raters. Moreover, future research could record eye movements to track the attentional spotlight of interlocutors with high temporal resolution, enabling the construction of dynamic saliency maps of the face. This approach would allow for the analysis of brief “thin-slice” segments of interaction, offering a direct parallel between computational and human judgments and helping to identify the combination of action units most informative for socially grounded classification.

Furthermore, machine classification in the present study was based on the activity of three facial action units implicated in social and emotional processes^24, 58, 59, 60^. While other action units also convey emotionally relevant information^61^, our model was limited to three data channels due to the constraints of the pre-trained convolutional neural network. An important direction for future research will be to explore whether additional action units could enhance machine learning based classification performance. However, increasing the number of action units may raise two issues. The first issue is overfitting, which can arise due to the higher dimensionality of the inputs. Another concern is the necessity to retrain the pre-trained CNNs to effectively process inputs with more than three channels that will require larger datasets.

The social context used in the present study — Zoom online meetings — offered significant advantages for the automated scoring of facial action unit activity. Participants’ alignment with the camera was facilitated, as evidenced by the high proportion of frames with excellent face detection scores. However, social interaction dynamics in virtual settings may differ from face-to-face interactions. Research suggests that the availability of nonverbal cues and facial expressiveness is reduced^62, 63^. Nevertheless, virtual meetings still transmit sufficient cues to support mimicry of subtle nonverbal behaviors^64^, and the present findings align with these observations. Future research could build on these findings by incorporating data from face-to-face interactions and a broader range of conversation topics, thereby enhancing our understanding of nonverbal dynamics across different social contexts.

## Conclusion

This study examined whether facial behavior alone, independent of linguistic content, encodes structured information about conversational context. Both human observers and a convolutional neural network (CNN) successfully classified conversation types based on facial dynamics, indicating that temporal facial patterns convey context-specific signals. These findings support the view that facial expressions are context-sensitive and dynamically aligned with interactional goals. They also highlight the utility of computational approaches for analyzing nonverbal behavior. Future work should extend these methods to multimodal models and more ecologically diverse datasets to better capture the complexity of real-world social interaction.

## Supplementary Information

Below is the link to the electronic supplementary material.

Supplementary Material 1

## Data Availability

Deidentified participant data and analysis code is publicly available under a CC BY 4.0 license here: https://doi.org/10.48606/nf7gwyrr2pe9nmq6.
