# Supplementary material for: Social context shapes facial dynamics: human and machine decoding of conversation topics
Source: Sci Rep. 2026 Jan 22;16:3059. doi: 10.1038/s41598-025-30403-9 (PMC12830719; doi:10.1038/s41598-025-30403-9)
Supplement: Supplementary file 1 — Supplementary Material 1 [file 41598_2025_30403_MOESM1_ESM.pdf]

Supplementary Information:

Social Context Shapes Facial Dynamics: Human and Machine Decoding of Conversation Topics

Prasetia Putra[1,2,*,+], Johanna Köchling[1,2,*,+], Jana Straßheim[1,2], Christophe Bousquet[1,2], Britta Renner[1,2], Harald Schupp[1,2]

[1] Centre for the Advanced Study of Collective Behaviour, University of Konstanz, Germany,
[2] Department of Psychology, University of Konstanz, Konstanz, Germany

[+] These authors contributed equally: Prasetia Putra and Johanna Köchling

* corresponding authors. Prasetia Putra (prasetia.putra@uni-konstanz.de) & Johanna Köchling (johanna.koechling@uni-konstanz.de)

This PDF file includes:

Supplementary Methods 1. **Sample Size Estimation**

Supplementary Figure 1. **Bayesian p-value Estimation for Study 1 (a) and 2 (b).**

Supplementary Methods 2. **Binary Classification of Conversation Topics Based on Descriptive Measures**

Supplementary Table 1. **Summary of Descriptive Statistics for Action Units 4, 6, and 12.**

Supplementary Table 2. **Classification Performance of the Logistic Regression Model.**

Supplementary Table 3. **Classification Performance of the Random Forest Model.**

Supplementary Methods 3. **Machine Learning-Based Classification of Conversation Topic: Ablation Studies**

Supplementary Table 4: **Classification Performance of Ablation Studies.**

Supplementary Methods 1. Sample Size Estimation

Study 1: We assumed a medium effect size (d = 0.5) and targeted ≈80% statistical power, defined as the proportion of simulations yielding a p-value below 0.001. Since each video was rated multiple times, we accounted for repeated measures by assuming five observations per video. As illustrated in Figure S1a, our sample size had adequate statistical power.

Study 2: Prior research suggests that a machine learning algorithm typically requires an effect size of 0.5 to achieve robust accuracy in binary classification (Rajput, Wang, & Chen, 2023). Using the triad as the unit of analysis, we simulated datasets ranging from 5 to 50 conversations. Based on Bayesian estimation, the simulations indicated that at least 15 sessions were needed for the median posterior p-value to fall below 0.01, and approximately 20 sessions for it to fall below 0.001.

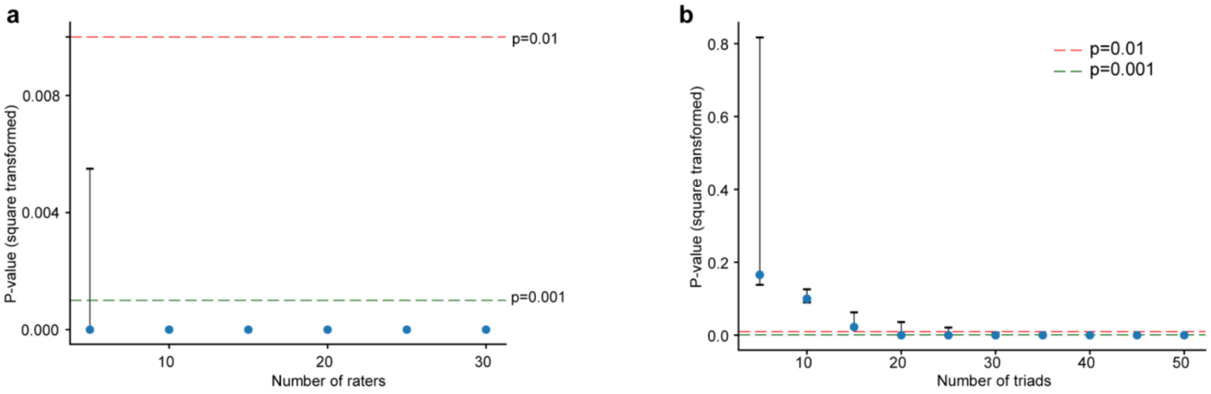

Supplementary Figure 1. **Bayesian p-value Estimation for Study 1 (a) and 2 (b).** Circles show the posterior median, with error bars indicating the 95% highest density interval. Please note, numbers for error bars were too small in (a) for 10 and more raters to be visible in the graph.

Supplementary Methods 2. Binary Classification of Conversation Topics Based on Descriptive Measures

Control analyses were conducted using conventional features derived from facial Action Unit (AU) activity. In total, we extracted eight features capturing the magnitude and temporal characteristics of AU activation. Three features characterized the overall activity level of each AU activity: (1) maximum, (2) mean, and (3) standard deviation. Five additional features quantified discrete episodes of AU activation. To this end, we discretized the continuous AU magnitude streams by applying Otsu's thresholding method (Otsu,1979) separately to AUs 4, 6, and 12 for each participant. Frames with AU magnitudes exceeding the threshold were considered active, and consecutive active frames were grouped into a single episode. Episodes shorter than 120 ms were excluded as artifacts; this threshold was empirically determined through pilot analyses, as higher thresholds led to reduced classification accuracy. For each participant's AU stream, we computed the (4) maximum episode duration, (5) mean episode duration, and (6) minimum episode duration. Additionally, (7) the total number of episodes was defined as the count of discrete activation periods per AU, and (8) the total number of active frames—expressed as the proportion of frames exceeding the threshold—captured the frequency of AU activation. The distributions of these parameters for the three action units, aggregated across participants in each condition, are shown in Supplementary Table 1.

Supplementary Table 1. Summary of Descriptive Statistics for Action Units 4, 6, and 12.

	Get-to-Know			Moral Dilemma		
	**AU 04**	**AU 06**	**AU 12**	**AU 04**	**AU 06**	**AU 12**
Activity - Max	0.58	0.66	0.69	0.58	0.66	0.69
Activity - M	0.39	0.50	0.49	0.38	0.47	0.51
Activity - SD	0.05	0.07	0.09	0.05	0.08	0.09
Duration - Max	83.04	900.05	186.49	54.74	318.02	315.07
Duration - M	4.36	709.07	22.14	4.81	22.83	51.00
Duration - Min	373.55	1739.81	1397.88	388.45	1323.26	1464.52
Activity - Total	119.26	153.00	157.21	119.07	137.86	161.81
Frames - Active	0.26	0.63	0.61	0.24	0.53	0.65

Note. This table displays the mean value calculated from the computed values for each participant within the condition.

Using these eight features as input, we trained logistic regression (linear) and random forest (non-linear) classifiers to assess conversation classification performance. The models were trained using the same procedure as the proposed deep learning approach, with 5-fold cross-validation.

To ensure comparability with the main findings reported in the manuscript, these analyses were conducted on speech-free segments of the conversation. Classification performance of the logistic regression and random forest models based on descriptive features of action unit activity was low. Using three action units as input, logistic regression achieved an accuracy of 60.71%, while Random Forest reached a comparable accuracy when relying solely on AU 12.

Supplementary Table 2. Classification Performance of the Logistic Regression Model.

Action Units	ACC (%) [CI 95%]	MCC [CI 95%]	AUC [CI 95%]
04	39.29 [37.29, 41.29]	-0.22 [-0.27, -0.17]	0.26 [0.24, 0.28]
06	53.57 [50.57, 56.57]	0.07 [0.01, 0.13]	0.45 [0.43, 0.47]
12	57.14 [53.14, 61.14]	0.14 [0.07, 0.21]	0.55 [0.53, 0.57]
04, 06, 12	60.71 [57.74, 63.69]	0.22 [0.16, 0.28]	0.53 [0.51, 0.55]

Note. The table reports classification metrics, including accuracy (ACC), Matthews correlation coefficient (MCC), and area under the curve (AUC), each presented with 95% confidence intervals.

Supplementary Table 3. Classification Performance of the Random Forest Model.

Action Units	ACC (%) [CI 95%]	MCC [CI 95%]	AUC [CI 95%]
04	50.00 [46.00, 54.00]	0.00 [-0.07, 0.07]	0.45 [0.43, 0.47]
06	50.00 [47.00, 53.00]	0.00 [-0.06, 0.06]	0.60 [0.58, 0.66]
12	60.71 [57.71, 63.71]	0.21 [0.15, 0.27]	0.57 [0.55, 0.63]
04, 06, 12	42.86 [39.86, 45.86]	-0.15 [-0.21, -0.09]	0.45 [0.43, 0.51]

Note. The performance of the Random Forest classifier decreased when using features from three AUs compared to using a single AU, likely due to overfitting,

Supplementary Methods 3. Machine Learning-Based Classification of Conversation Topic: Ablation Studies

To provide preliminary insight into the contribution of individual action units (AUs) to classification performance, we conducted ablation studies using fewer than three AUs. Classification performance based on a single AU was clearly inferior. Combinations of two AUs improved performance but remained less accurate than models using all three AUs. As noted, our model was limited to three AUs, so we were unable to assess whether including additional AUs would further enhance classification accuracy.

Supplementary Table 4. Classification Performance of Ablation Studies.

Action Units	ACC (%) [CI 95%]	MCC [CI 95%]	AUC [CI 95%]
04	57.14 [54.14, 60.14]	0.15 [0.09, 0.21]	0.58 [0.56, 0.60]
06	67.86 [65.86, 69.86]	0.36 [0.31, 0.41]	0.78 [0.77, 0.79]
12	67.86 [65.86, 69.86]	0.36 [0.31, 0.41]	0.70 [0.68, 0.72]
04, 06	78.57 [76.57, 80.57]	0.58 [0.54, 0.62]	0.80 [0.79, 0.81]
04, 12	71.43 [68.43, 74.43]	0.43 [0.37, 0.49]	0.70 [0.68, 0.72]
06, 12	64.29 [62.29, 66.29]	0.29 [0.24, 0.34]	0.68 [0.66, 0.70]
04, 06, 12*	82.14 [80.14, 84.14]	0.64 [0.59, 0.69]	0.90 [0.89, 0.91]

Note. Results from the main analyses were included to enable direct comparison across conditions (marked with *).

References Supplement

Otsu, N. (1979). A Threshold Selection Method from Gray-Level Histograms. *IEEE Transactions on Systems, Man, and Cybernetics*, *9*(1), 62–66. https://doi.org/10.1109/TSMC.1979.4310076

Rajput, D., Wang, W.-J., & Chen, C.-C. (2023). Evaluation of a decided sample size in machine learning applications. *BMC Bioinformatics*, *24*(1). https://doi.org/10.1186/s12859-023-05156-9
